# The regulation of TGF-β/SMAD signaling by protein deubiquitination

**DOI:** 10.1007/s13238-014-0058-8

**Published:** 2014-04-23

**Authors:** Juan Zhang, Xiaofei Zhang, Feng Xie, Zhengkui Zhang, Hans van Dam, Long Zhang, Fangfang Zhou

**Affiliations:** 1 Life Sciences Institute, Zhejiang University, Hangzhou, 310058 China; 2 Department of Molecular Cell Biology, Cancer Genomics Centre Netherlands and Centre of Biomedical Genetics, Leiden University Medical Center, Postbus 9600, 2300 RC Leiden, The Netherlands

**Keywords:** TGF-β, TβRI, SMAD, DUB, ubiquitin, deubiquitination

## Abstract

Transforming growth factor-β (TGF-β) members are key cytokines that control embryogenesis and tissue homeostasis via transmembrane TGF-β type II (TβR II) and type I (TβRI) and serine/threonine kinases receptors. Aberrant activation of TGF-β signaling leads to diseases, including cancer. In advanced cancer, the TGF-β/SMAD pathway can act as an oncogenic factor driving tumor cell invasion and metastasis, and thus is considered to be a therapeutic target. The activity of TGF-β/SMAD pathway is known to be regulated by ubiquitination at multiple levels. As ubiquitination is reversible, emerging studies have uncovered key roles for ubiquitin-removals on TGF-β signaling components by deubiquitinating enzymes (DUBs). In this paper, we summarize the latest findings on the DUBs that control the activity of the TGF-β signaling pathway. The regulatory roles of these DUBs as a driving force for cancer progression as well as their underlying working mechanisms are also discussed.

## INTRODUCTION

Protein ubiquitination is a reversible process. Deubiquitinating enzymes (DUBs) function to remove covalently conjugated ubiquitins from their target proteins to regulate substrate activity and/or abundance (Nijman et al., 2005). DUBs have amongst others been implicated in cellular signaling pathways that control cell proliferation and differentiation. TGF-β/SMAD signaling can play a tumor promoting role in advanced cancer and certain essential components of this pathway, TGF-β receptors and SMADs are known to be downregulated via protein ubiquitination by E3 ligases (Massague, 2008a). Multiple DUBs have been shown to target ubiquitinated TGF-β/SMAD signaling components and to be associated with high risk for cancer metastasis, both in animal models and in clinical analysis (Eichhorn et al., 2012; Inui et al., 2011; Zhang et al., 2012a, b). As the DUBs are druggable proteins, these studies may provide possibilities for novel and effective therapeutic treatments (Cohen and Tcherpakov, 2010; Hoeller and Dikic, 2009). This paper revisits the signal transduction mechanisms and biological features of TGF-β/SMAD pathways, followed by an overview of the ubiquitination regulation of the TGF-β/SMAD pathways by ubiquitination and a brief introduction of the human DUB family. It finally highlights the newly identified DUB members acting on TGF-β/SMAD signaling as well as their emerging roles in the regulation of cancer invasion and metastasis.

## TGF-β SIGNALING

### Signaling pathways induced by the transforming growth factor-β superfamily

The TGF-β superfamily contains a number of structurally and functionally related secreted cytokines. Since TGF-β was discovered in 1983 (Frolik et al., 1983), more than 30 members of this family have been identified and verified. Members of the TGF-β family are characterized by the highly conserved cysteine residues, also known as the cystine knot (CK) motif (Galat 2011). According to the sequences similarities and their distinct downstream signaling pathways, the TGF-β superfamily can be divided into several subfamilies, including TGF-βs, bone morphogenetic proteins (BMPs), nodal, growth and differentiation factors (GDFs), Müllerian inhibitory factor (MIF), activins and inhibins (Massaous and Hata, 1997). Although different TGF-β members have distinct cellular functions, they all act on cells as dimers.

The TGF-β family members bind to the type I and type II serine/threonine kinase receptors on the cell surface. The serine/threonine kinase receptor family contains twelve members, that are seven type I receptors, also known as activin receptor-like kinases (ALKs), and five type II receptors (Huang et al., 2011; Massague, 2008b). Both type I and type II receptors are expressed ubiquitously in mammalian cells.

The canonical intracellular signaling induced by TGF-β ligands is mediated by SMAD family proteins. Based on their function differences, the SMAD family is divided into three groups, that are receptor-associated SMADs (R-SMADs), co-operating SMADs (Co-SMADs), and inhibitory SMADs (I-SMADs) (Ross and Hill, 2008). Only R-SMADs are targeted for activation via phosphorylation by the active type I receptor kinase. In general, diverse TGF-β ligand binds to and activates a characteristic combination or combinations of different type I and type II receptors on the plasma membrane, and targets specific R-SMADs for activation. Upon TGF-β-induced receptor complex formation, TβRII kinase phosphorylates TβRI, e.g. ALK5, on specific serine and threonine residues in its juxtamembrane. Subsequently, the activated ALK5 induces the phosphorylation of the R-SMADs SMAD2 and SMAD3, which can form heteromeric complexes with the Co-SMAD SMAD4. SMAD2/3 can be activated by TGF-βs, activins, and nodal upon complex formation between ALK4/5/7 and TGF-β type II receptor (TβRII) and activin receptor 2 (ACVR2). SMAD1/5/8, can be activated by BMP ligands through complex formation between the type I receptor ALK1/2/3/6 and BMP type II receptor (BMPRII) or ACVR2. The Co-SMAD SMAD4 functions as a central transducer in the TGF-β responses. The two I-SMADs, SMAD6 and SMAD7, enable tight control of TGF-β signaling through negative regulation: they can compete with Co-SMAD for the interaction with the phosphorylated R-SMADs and they can recruit SMURF E3 ubiquitin ligase to the type I receptors (Itoh and ten Dijke, 2007; Kavsak et al., 2000).

**Figure 1:**
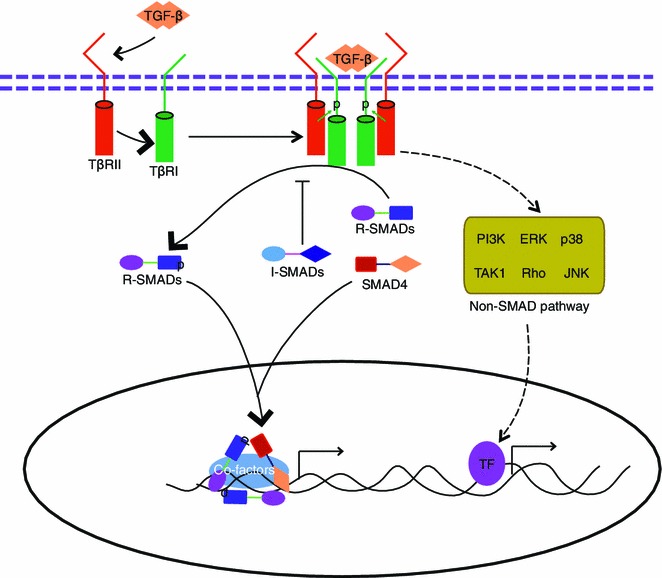
**A schematic representation of the TGF-β signaling pathway**. Upon ligand binding, the TGF-β type II receptor (TβRII) recruits and phosphorylates the type I receptor (TβRI) in the cell membrane, and then the activated type II-I heteromeric receptor complex can induce phosphorylation of R-SMADs. Activated R-SMADs can recruit Co-SMAD (SMAD4) for binding. The R-SMAD/Co-SMAD complexes translocate and accumulate in the nucleus and then initiate the expression of the target transcription factors with the help of other co-factors. TGF-β can in addition promote other intracellular (non-SMAD) signaling pathways, such as mitogen activated protein kinases (MAPKs) pathways, the phosphoinositide 3-kinase (PI3K)/AKT pathway, the nuclear factor κB (NF-κB) pathway, and the Rho-like GTPase pathway

The heteromeric SMAD complexes formed by the activated R-SMADs and SMAD4 accumulate in the nucleus, where they regulate target gene expression (Fig. 1) (Heldin et al., 1997). In addition to this canonical SMAD-dependent TGF-β signaling pathway, there are other non-SMAD pathways that can be activated by the TGF-β receptors via either phosphorylation or direct interaction. These non-SMAD pathways include various branches, such as mitogen activated protein kinases (MAPKs) pathways, phophoinositde 3-kinase (PI3K)/Akt pathways, nuclear factor κB (NF-κB) pathways, and Rho-like GTPase pathways (Fig. 1) (Derynck and Zhang, 2003; Mu et al., 2012; Sanchez-Elsner et al., 2001; Zhang, 2009).

### Functions of TGF-β signaling

TGF-β/SMAD signaling is multifunctional in regulating cell growth, differentiation, apoptosis, migration and invasion/metastasis (Goumans and Mummery, 2000; Hogan, 1996; Massague et al., 2000; Proetzel et al., 1995; Sanford et al., 1997; Schier, 2003; Whitman, 1998). Disturbances of TGF-β/SMAD signaling are widely shown to be involved in human diseases, including hereditary hemorrhagic telangiectasia, fibrosis diseases, atherosclerosis, hereditary synostosis, hereditary chondrodysplasias, cleidocranial dysplasia and familial primary pulmonary hypertension (Blobe et al., 2000; Massague et al., 2000). In human cancer, TGF-β/SMAD signaling can have a dual role. In the early phase of tumor progression, TGF-β/SMAD plays a tumor suppressing role (Massague et al., 2000). On the contrary, TGF-β/SMAD can promote advanced tumor progression such as tumor cell invasion, dissemination/metastasis, and immune evasion (Massague, 2008a). Thus the functional outcome of the TGF-β response is context-dependent and determined both by cell, tissue, and cancer types.

TGF-β signaling inhibits cell proliferation in a multitude of cell types, including normal endothelial, epithelial, hematopoietic, and neural cells, certain types of mesenchymal cells, and especially many primary cancer cells (Massague et al., 2000). TGF-β can downregulate the c-Myc oncogene levels thereby counteracting Myc-induced cell proliferation via upregulation of cyclins and downregulation of p21 (also known as WAF1) (Dang, 1999; Warner et al., 1999). TGF-β can also induce growth arrest by its inhibitory role on cyclin-dependent kinases (CDK) via upregulation of p15 (also termed as INK4B) and p21 expressions and downregulation of CDC25A expression (Claassen and Hann, 2000; Iavarone and Massague, 1997). The tumor suppressing role of TGF-β/SMAD pathway seems particularly critical in the gastro-intestinal tract, since large subsets of pancreatic, gastric, and colon cancers carry mutations or deletions in TGF-β receptors or SMADs (Grady et al., 1999; Markowitz et al., 1995; Myeroff et al., 1995; Parsons et al., 1995; Schutte et al., 1996; Hahn et al., 1996; Schutte et al., 1996; Yakicier et al., 1999).

Advanced cancers such as gliomas, breast and prostate cancers usually do not acquire mutations in the core components of TGF-β/SMAD signaling, but can bypass the TGF-β/SMAD tumor-suppressive arms through other, more downstream (epi)genetic changes, allowing the tumor promoting arm of TGF-β/SMAD signaling to actively drive tumor cell progression (Jennings and Pietenpol, 1998; Jones et al., 2009; Takenoshita et al., 1998; Vincent et al., 1996; Xu et al., 2009). Tumors with such signatures are resistant to TGF-β/SMAD mediated growth arrest but can undergo epithelial-to-mesenchymal transition (EMT) and invasion/metastasis. EMT is a process required for embryonic development and wound healing, but is employed by tumor cells to invade normal tissue and/or spread to distant organs. During EMT, carcinoma cells lose cell polarity and cell-cell contacts, and acquire fibroblastic-like properties as evidenced by morphological observations at the invasive fronts of human tumors (Kalluri and Weinberg, 2009; Katsuno et al. 2012). The TGF-β/SMAD pathway is a critical regulator of EMT in development *in vivo* (Kaartinen et al., 1995). In tumor cells, SMAD3/SMAD4 mediates transcription of *SNAIL* and *SLUG*, two master regulators of the EMT process (Miyazono, 2009; Naber et al. 2013). TGF-β/SMAD signaling also strongly drives the appearance of various molecular hallmarks of cells undergoing EMT, such as the decreased expression of epithelial cell-cell junction proteins including E-cadherin and zona occludens 1 (ZO-1), and at the same time it can induce the expression of mesenchymal markers, such as N-cadherin, vimentin, α-smooth muscle actin (α-SMA), and fibronectin (Heldin et al., 2009; Huber et al., 2005; Moustakas and Heldin, 2007; Xu et al., 2009).

The TGF-β induced pathways also can enable advanced invasive tumor cells to disseminate to other organs and form metastatic lesions (Bos et al., 2010; Nguyen et al., 2009). TGF-β stimulated metastatic dissemination is typically studied in bone and lung metastases of breast and prostate tumors. For instance, the SMAD3/SMAD4 complex was found to mediate the induction of connective tissue growth factor (*CTGF*) and interleukin (*IL-11*), which are critical factors for bone metastasis (Deckers et al., 2006; Kang et al., 2005; Kang et al., 2003; Petersen et al., 2010). By inducing angiopoietin-like 4 (ANGPTL4), TGF-β primes dissemination towards the lung (Padua et al., 2008). An increasing amount of studies provide evidences that the TGF-β/SMAD pathway is widely involved in multiple processes of cancer metastasis, including early invasion, intravasation, and later extravasation and colony formation (Drabsch and ten Dijke, 2012).

## UBIQUITINATION AND ITS ROLE IN TGF-β SIGNALING

### Ubiquitin and ubiquitination

Ubiquitin is a small regulatory protein (76 amino acids) that exists in almost all kinds of eukaryotic cells. Ubiquitin has originally been characterized as a covalently attached signal for ATP-dependent proteasomal degradation of substrate proteins (Hershko and Ciechanover, 1998), although it also plays a role in both the lysosomal and autophagic degradation pathways (Clague and Urbe, 2010). In addition to the protein degradation pathways, ubiquitin attachment is also implicated in dynamic cellular events, such as the transduction of cellular signals, gene transcription as well as DNA damage and repair (Hunter, 2007; Jackson and Durocher, 2013). Ubiquitin contains seven lysine residues in its sequence and each of them allows polyubiquitin chain conjugation via a covalently linking to the carboxyl end of another ubiquitin (Pickart and Eddins, 2004).

**Figure 2:**
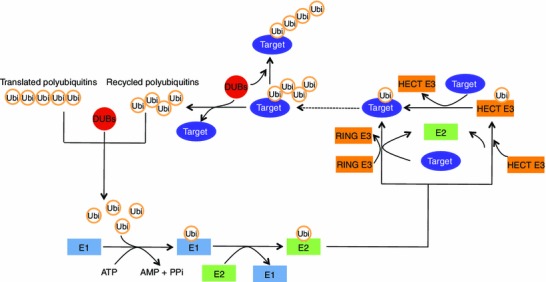
**An overview of ubiquitination and deubiquitination processes**. The conjugation process of ubiquitin to the substrate proteins normally requires three steps: a) the initial step is to activate the C-terminus of the ubiquitin protein by an E1 and this process requires ATP, b) the intermediate step is to transfer ubiquitin from the E1 and conjugate to an E2, c) the final step is to covalently conjugate the ubiquitin to the substrate protein which normally facilitated by an E3 enzyme. DUBs can catalyse the removal of ubiquitin from the conjugated substrates and also generate free ubiquitins from the newly translated polyubiquitins or recycle ubiquitins after the polyubiquitinated protein substrates are degraded (For further details see text)

Ubiquitination is an enzymatic and post-translational modification process involving covalently linking of one ubiquitin (monoubiquitination) or more ubiquitins (polyubiquitination) to the substrate protein. The conjugation process of ubiquitin to the substrate normally requires three steps: a) the initial step is to activate the C-terminus of the ubiquitin protein by a ubiquitin-activating enzyme (E1), b) the intermediate step is to transfer and conjugate ubiquitin from the E1 enzyme and conjugate to an ubiquitin-conjugating enzyme (E2), c) the last step is to covalently conjugate the ubiquitin protein to the substrate protein which is normally facilitated by a substrate-specific ubiquitin ligase (E3) (Fig. 2) (Dikic, 2009; Pickart and Eddins, 2004; Schwartz and Ciechanover, 2009; Weissman, 2001). Two types of E3 ligases can facilitate this last step: one group of E3 ligases carries an E6-AP carboxyl terminus (HECT) domain, via which the E2 ligase can transfer the ubiquitin to the final substrate protein, the other group is characterized by a so-called really interesting new gene (RING) domain that may help to transfer E2-ubiquitin to the protein substrate (Dikic, 2009; Pickart and Eddins, 2004; Schwartz and Ciechanover, 2009; Weissman, 2001). Ubiquitination can alter the activity or localization of the substrate protein (mainly in case of monoubiquitination), target substrate proteins for degradation, or allow proteins to function as a scaffold (mainly via polyubiquitination) (Pickart and Eddins, 2004). In the case of polyubiquitination, there are at least 8 different types of poly ubiquitins linkages (Lysine-6, Lysine-11, Lysine-27, Lysine-29, Lysine-33, Lysine-48 and Lysine-63 polyubiquitination, and linear ubiquitination) can exist in the cells (Dikic, 2009; Weissman, 2001). Importantly different types of polyubiquitination linkages dictate distinct functions. For example, poly ubiquitins linked with Lysine-48 provide the main targeting signals for proteasomal degradation, whereas polyubiquitins linked with Lysine-63 enable the substrate protein to function as scaffolds to recruit other partners and thereby to participate in multiple cell processes, such as kinase activation, DNA repair, and protein synthesis (Schwartz and Ciechanover, 2009).

### Ubiquitination regulation in TGF-β signaling

Ubiquitination modifies a series of TGF-β pathway components, including receptors, R-SMADs, Co-SMAD, I-SMADs, and their regulators, via different E3 ubiquitin ligases (Inoue and Imamura, 2008). TβRI can be polyubiquitinated by SMAD-ubiquitination-related factor (SMURF) 1/2, WW domain-containing protein 1 (WWP1) and neural precursor cells-expressed developmentally down-regulated 4 (NEDD4)-2 with the help of the inhibitory SMAD7 (Ebisawa et al., 2001; Kavsak et al., 2000; Komuro et al., 2004; Kuratomi et al., 2005). This alters receptor stability on the membrane as well as the receptor internalization/endocytosis status and thus tightly restricts sensitivity of cells towards TGF-β stimulation. SMAD protein stability is also controlled by polyubiquitination. SMAD1 can be polyubiquitinated by SMURF1/2 and carboxyl terminus of Hsc70-interacting protein (CHIP) (Li et al., 2004; Zhang et al., 2001; Zhu et al., 1999). SMAD2 is reported to be polyubiquitinated by SMURF2, NEDD4L, or WWP1 (Kuratomi et al., 2005; Lin et al., 2000; Seo et al., 2004). SMAD3 is polyubiquitinated by CHIP (Xin et al., 2005). Phosphorylated SMAD2/3 can be polyubiquitinated by ARKADIA after the target gene transcription is initiated (Mavrakis et al., 2007). SMAD7 is shown to be targeted for polyubiquitination by ARKADIA and RNF12 (Koinuma et al., 2003; Liu et al., 2006; Zhang et al., 2012a; Zhang et al., 2012b). Similar to R-SMADs, SMAD4 could also be polyubiquitinated by the HECT domain ubiquitin E3 ligases SMURFs, WWP1, or NEDD-2 (Moren et al., 2005). Besides TGF-β receptors and SMADs, other key regulators of TGF-β signaling pathway can also be polyubiquitinated for degradation. As negative regulator of the TGF-β pathway, SNON is polyubiquitinated and targeted for degradation by SMURF2 or anaphase-promoting complex (APC) (Bonni et al., 2001; Stroschein et al., 2001).

In addition to activation of (canonical) signaling via the SMADs, the TGF-β receptor complex can also recruit TNF receptor-associated factor (TRAF) 4 and TRAF6, which then by K63-polyubiquitination activates the effector kinase TGF-β-activated kinase 1 (TAK1); TAK1 subsequently phosphorylates MAPK kinases, leading to activation of p38 or JNK (Sorrentino et al., 2008; Yamashita et al., 2008; Zhang et al., 2013a). Moreover, recent studies reveal a critical function for monoubiquitination on SMADs. The transcriptional activity of SMAD4 was shown to be antagonized upon monoubiquitination by Ectodermin/TRIM33/TIF1γ (Dupont et al., 2009). Similarly, monoubiquitination of R-SMADs triggered by SMURF has been shown to exert an inhibitory role (Inui et al., 2011; Tang et al., 2011). In addition, conjugation of a single ubiquitin molecule conjugation to SMAD6 by the E2 enzyme UBE2O appears already sufficient to attenuate the inhibitory function of SMAD6 on BMP signaling (Zhang et al., 2013b).

## Deubiqutination and human dub family members

### Deubiquitination

Ubiquitination is a reversible modification process and is counteracted by a process termed deubiquitination. Deubiquitination involves the removal of ubiquitin from its conjugates by deubiquitinating enzymes/deubiquitinases (DUBs) (Amerik and Hochstrasser, 2004; Nijman et al., 2005). DUBs are a large group of proteases that cleave ubiquitins from proteins (Nijman et al., 2005). DUBs also assist to generate free molecules from the newly translated polyubiquitins and recycle ubiquitins after the polyubiquitinated protein substrates are degraded (Fig. 2) (Komander et al., 2009). Therefore, DUBs play key roles in the regulation of signal transduction by controlling ubiquitin homeostasis thereby affecting the stability, activity and/of subcellular localization of proteins (Komander et al., 2009).

### The human DUB family

**Figure 3:**
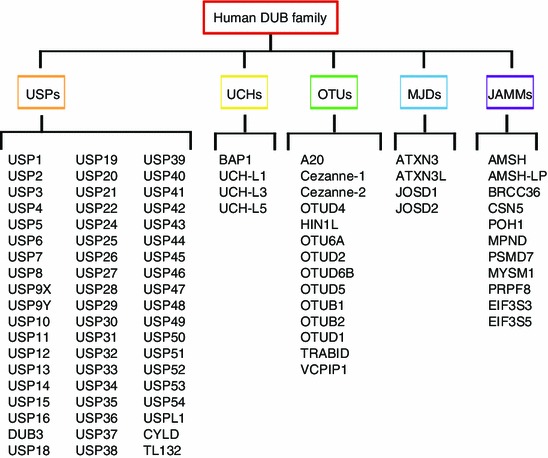
**A schematic summary of human DUB family**. The DUB family can be divided into five subfamilies, including ubiquitin-specific proteases (USPs, 57 members), ubiquitin C-terminal hydrolases (UCHs, 4 members), ovarian tumor proteases (OTUs, 14 members), Machado-Joseph disease proteases (MJDs, 4 members), and JAB1/MPN/Mov34 proteases (JAMMs, 11 members)

The human genome encodes almost 90 DUBs of which 79 are predicted to be active. According to the sequence similarity and the possible functions, the DUBs family can be divided into 5 subfamilies, including ubiquitin-specific proteases (USPs), ubiquitin C-terminal hydrolases (UCHs), ovarian tumor proteases (OTUs), Machado-Joseph disease proteases (MJDs), and JAB1/MPN/Mov34 proteases (JAMMs) (Fig. 3) (Komander et al., 2009; Nijman et al., 2005; Reyes-Turcu et al., 2009).

### Functions of DUBs

Since polyubiquitination can serve as a tag for protein destruction thus allows DUB mediated deubiquitination of such polyubiquitinated proteins will promote protein stability. USP1 for instance stabilizes inhibitors of DNA binding proteins (IDs) through deubiquitination (Williams et al., 2011). HAUSP (also termed as USP7) deubiquitinates p53, and is therefore considered to be an important positive regulator of p53 stabilization (Li et al., 2002). USP28 is overexpressed in colon and breast tumors, and by counteracting the ubiquitination activity of SCF-Fbxw7 ligase it causes the stabilization of cyclin E1 and c-Myc (Popov et al., 2007a; Popov et al., 2007b). Similarly, USP33 promotes centrosome biogenesis via specific and potent stabilization of the centriolar coiled coil protein CP110 (Li et al., 2013), and USP22 has been found to strengthen the NAD-dependent histone deacetylase Sirt1 to antagonize p53 activation (Lin et al., 2012).

Conceptually, removal of monoubiquitination by DUBs should also reverse for instance substrate localization or substrate-induced transcription activation in case monoubiqutination of the substrate has theses effects (see previous chapter). Indeed, such effects were reported for HAUSP-mediated ubiquitin removal of PTEN (phosphatase and tensin homologue deleted in chromosome 10) and FOXO (Forkhead box O) 4 (Song et al., 2008; van der Horst et al., 2006).

Another important function of DUBs is exemplified by their capability to reverse the non-degradative polyubiquitin chain conjugation on central signaling molecules. For instance, AMSH and AMSH-LP promote receptor trafficking by specifically cleaving Lysine-63 linked polyubiquitin chains from internalized receptors (McCullough et al., 2004; Sato et al., 2008) and the deubiquitinases CYLD, A20 and USP4 antagonize Lysine-63 polyubiquitin chain conjugation on TRAF6, thereby disrupting the docking sites for downstream innate immune signaling activation (Boone et al., 2004; Brummelkamp et al., 2003; Deng et al., 2000; Kovalenko et al., 2003; Trompouki et al., 2003; Turer et al., 2008; Xiao et al., 2012; Zhang et al., 2012a; Zhang et al., 2012b). Similarly, linear polyubiquitin chain formation on NF-κB essential modulator (NEMO) by the E3 ligase linear ubiquitin chain assembly complex (LUBAC) is cleaved by CYLD and more specifically by OTULIN (also termed as FAM105B) (Gerlach et al., 2011; Ikeda et al., 2011; Keusekotten et al., 2013; Niu et al., 2011; Rivkin et al.^2013^; Tokunaga et al., 2009). In the Wnt signal transduction pathway, CYLD inhibits β-catenin signaling by removing Lysine-63 linked ubiquitination from Dishevelled (Tauriello et al., 2010). Moreover, nuclear functions of DUBs in transcription and RNA processing have been uncovered (Clague et al., 2012). In this article, we will further focus on recent advances that help to understand the role of DUBs in TGF-β/SMAD signaling.

## FUNCTIONAL DUBS IN TGF-β SIGNALING

Unlike the regulation of TGF-β signaling by ubiquitination, which has been intensely studied for the last decades, the role of DUB-mediated deubiquitination in the TGF-β signaling pathway is only recently emerging. It is until recently that a few reports just unveil this mystery in which several functional DUBs have now been identified and found to be potent TGF-β/SMAD modulators (Table 1) (Al-Salihi et al., 2012; Dikic, 2009; Eichhorn et al., 2012; Schwartz and Ciechanover, 2009; Wicks et al., 2005; Zhang et al., 2012a; Zhang et al., 2012b; Zhao et al., 2011).

**Table 1:** **Summary of DUBs implicated in TGF-β signaling**

DUB	Targets (possible targets)	References
UCH37	Type I receptor	Wicks et al., 2005
USP4	Type I receptor	Zhang et al., 2012a, b
USP11	Type I receptor	Al-Salihi et al., 2012
USP15	Type I receptor; R-SMADs	Eichhorn et al, 2012; Inui et al., 2011
USP9X	SMAD4	Dupont et al., 2009
CYLD	SMAD7	Zhao et al., 2011
AMSH	(Binds to SMAD6)	Itoh et al., 2001
AMSH-2	(Binds to SMAD2 and SMAD7)	Ibarrola et al., 2004

### UCH37 as the first identified DUB in TGF-β/SMAD pathway

UCH37, a member of the UCH enzymes subfamily, and also known as UCHL5 in mouse, has been identified as a SMAD3-binding partner (Wicks et al., 2005). Previously, it was shown to function as a component of the 26S proteasome and thus might play a role in the editing of polyubiquitinated protein substrates (Weissman, 2001). UCH37 also interacts with SMAD7 through the SMAD7 N-terminal domain (1–260 aa), and not via the PY motif, a region that mediates SMAD7’s binding to SMURF (Wicks et al., 2005). Via SMAD7, UCH37 can further be recruited to TβRI, where it removes polyubiquitin chains synthesized by SMURF (Wicks et al., 2005).

### USP4 is a DUB for TGF-β type I receptor

**Figure 4:**
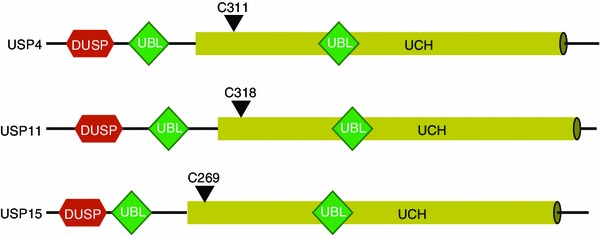
**Alignment of USP4 and its paralogs USP11 and USP15**. The highly similar domain structure of USP4, USP11, and USP15 is schematically illustrated; the degree of identity is also shown. Overall, USP4 shares 46.7% identity with USP11, and 59.6% identity with USP15. USP11 shares 45.9% identity with USP15 (For further details see text)

**Figure 5:**
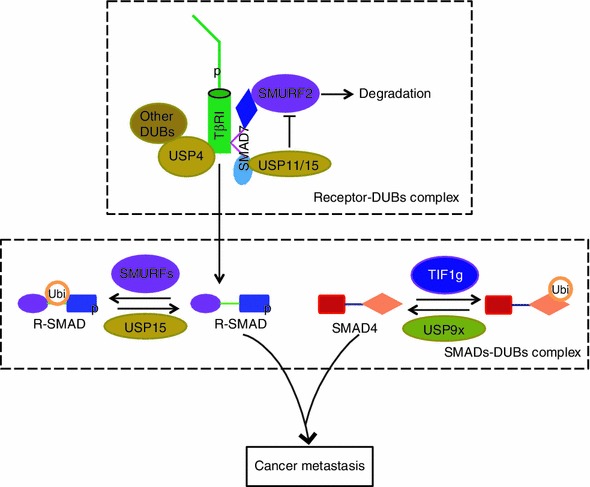
**Effects of USP4, USP15, and USP9X on TGF-β type I receptor and SMADs**. As depicted schematically, USP4 associates with and deubiquitinates TβRI and recruits other DUBs. USP15 binds TβRI via SMAD7 and competes with SMURF2 to balance TβRI ubiquitination. The transcriptional activity of R-SMAD can be restricted by the SMURFs via monoubiquitination and reversed by USP15. USP9X reverses SMAD4 monoubiquitination that can be conjugated by Ectodermin/TIF1γ E3 ligase

USP4, a member of USP subfamily, is the first deubiquiting enzymes that have been identified in mammalian cells. USP4 is a very stable protein as it can deubiquitinate itself (Wada and Kamitani, 2006). In the past year, gathered observations by several groups have revealed that USP4 is widely involved in multiple signaling pathways including the Wnt/β-catenin pathway, the innate immune response pathway, p53 signaling pathway and in particularly the TGF-β/SMAD signaling pathway (Liu et al., 2002; Xiao et al., 2012; Zhang et al., 2012a; Zhang et al., 2012b; Zhao et al., 2009). In a genome wide gain-of-function screen that covered nearly 27,000 genes, USP4, as well as USP11/USP15 were found to play a strong activating role in TGF-β/SMAD signaling. It is not so surprising USP4/11/15 share the ability to potentiate TGF-β/SMAD signaling because they share highly conserved domains and similarity in their protein sequences (Fig. 4). As to underlying mechanism USP4 was demonstrated to deubiquitinate and stabilize TβRI in the plasma membrane through direct association (Zhang et al., 2012a; Zhang et al., 2012b) (Fig. 5). A series of *in vitro* and *in vivo* experiments showed that USP4 is a critical and selective regulator of TGF-β/SMAD signaling in mammalian cells and zebrafish embryos. The fact that USP4 is highly expressed in various cancers indicated a critical role for USP4 in the tumor-promoting arm of the TGF-β/SMAD pathway. Indeed, analysis in malignant breast cancer cells revealed that USP4 could regulate TGF-β-induced EMT, migration *in vitro* and stimulate TGF-β/SMAD signaling-dependent breast cancer invasion and metastasis *in vivo* (Zhang et al., 2012a; Zhang et al., 2012b). Importantly, USP4 could bind to itself and also interact with USP11 and USP15, and thus may be part of a DUB complex when exerting its function (Zhang et al., 2012a; Zhang et al., 2012b). Interestingly, USP4 was found also to associate with AKT and to be phosphorylated by AKT on it conserved Ser445 motif. This phosphorylation promotes USP4 localization in membrane and cytoplasm, where USP4 deubiquitylates TβRI. This study suggests that Akt activation in breast cancer cells induces USP4 to relocate and stabilize TβRI in the plasma membrane, and thereby enforces TGF-β-induced pro-tumorigenic responses (Zhang et al., 2012a; Zhang et al., 2012b). Moreover, aberrant over-activation of PI3K/AKT pathway is frequently observed in human cancers and this could blunt tumor suppressing pathways. PI3K/AKT activation may thus redirect TGF-β intracellular signaling and thereby contribute to its switch from tumor suppressor to tumor promoter.

### USP11 is another DUB for TGF-β type I receptor

Like USP4, USP11 is involved in multiple signaling pathways. For instance, USP11 has been shown to associate with and stabilize RanGTP-associated protein RanBPM, BRCA2, HPV-16E7, nucleoprotein (Jennings and Pietenpol, 1998), and IκBα, depending on its DUB activity (Ideguchi et al., 2002; Liao et al., 2010; Lin et al., 2008; Schoenfeld et al., 2004; Sun et al. 2009). In a DUB activity independent manner, USP11 is involved in the IκB kinase α (IKKα)-p53 signaling pathway and also function in the regulation of DNA double-strand repair (Wiltshire et al., 2010; Yamaguchi et al., 2007). In addition to the USP4 study described above (Zhang et al., 2012a; Zhang et al., 2012b), an independent study identified USP11 as positive regulator of TGF-β signaling (Al-Salihi et al., 2012). In this study USP11 was identified by a proteomic approach in search for novel binding partners of TGF-β signaling components. USP11 was found to interact with SMAD7 and be recruited via SMAD7 to deubiquitinate TβRI and promote TGF-β signaling (Al-Salihi et al., 2012).

### USP15 is a DUB for both TGF-β type I receptor and R-SMADs

A DUB RNAi library mediated loss-of-function screen also identified USP15 as a key regulator of TGF-β signaling (Eichhorn et al., 2012). Distinct from USP4, USP15 was not found to bind to activate TβRI directly; rather, it is recruited to the active receptor by SMAD7. In the model proposed by the authors, SMAD7 acts as a scaffold that brings both the ubiquitin E3 ligase SMURF2 and the deubiquitinase USP15 to the TβRI receptor (Fig. 5). When the level of (active) TGF-β is low, TβRI ubiquitinylation by SMURF2 is quickly removed by USP15 according to this model. However, when TGF-β signaling is increased, a higher level of SMAD7 expression is induced as a feedback response and this will make the amount of USP15 insufficient, thereby limiting the duration of TGF-β/SMAD signaling (Aggarwal and Massague, 2012; Eichhorn et al., 2012).

As described above, advanced human cancer cells that retain TGF-β/SMAD signaling but lack tumor suppressive responses can make use of the SMAD pathway to their advantages, and via SMAD3/SMAD4 stimulate pro-invasive and pro-metastatic target genes (for example, *IL11*, *CTGF*, *CXCR4*) and reprogram (EMT) phenotypes (Aggarwal and Massague, 2012). This happens frequently in aggressive breast carcinoma and glioblastoma. In this respect it is important to note that while functional linkage of USP4 to the TGF-β/SMAD pathway was shown by employing a breast cancer model, USP15 can enhance the tumorigenic effect of TGF-β in glioblastoma (Eichhorn et al., 2012).

Results from an independent screen using an RNAi library against human DUB family members also indicated the physiological relevance of USP15 in regulating TGF-β superfamily function. In this study USP15 was found to potentiate both the TGF-β pathway and the related BMP pathway by targeting mono-ubiquitinated R-SMADs for deubiquitination (Fig. 5) (Inui et al., 2011). Thus, USP15 is not only required for TGF-β signal transduction and biological functions, including TGF-β-induced cell arrest and cell migration, but also necessary for BMP-induced osteoblast differentiations. Moreover, Xenopus embryo analysis in this study also uncovered a role for USP15 in embryonal development *in vivo*, dependent on its effect on TGF-β superfamily signaling (Inui et al., 2011).

### OTUB1 activates TGF-β signaling via activating (phospho-) SMAD2/3

Recently, OTU domain-containing ubiquitin aldehyde-binding protein 1 (OTUB1) was found to act on R-SMAD as well (Herhaus et al., 2013). However, different from USP15, OTUB1 enhances TGF-β signaling by inhibiting the ubiquitination and degradation of active SMAD2/3 (and not the inactive un-phosphorylated form), because the association of OTUB1 to SMAD2/3 is phosphorylation dependent. Moreover, OTUB1 was found to antagonize SMAD2/3’s ubiquitination independent of its catalytic activity as it interacts with E2 enzymes and inhibits efficient ubiquitin transfer from E2 to E3. This mechanism is reminiscent to the mechanism described in an earlier study on OTUB1-mediated inhibition of ubiquitination (Wiener et al., 2012).

### CYLD binds to Smad7

The deubiquitinase cylindromatosis (CYLD) was first identified as a tumor suppressor gene, mutations in patients with familial cylindromatosis (Bignell et al., 2000). As a member of USPs subfamily, CYLD can antagonize Lysine-63 polyubiquitin chain conjugation (Kovalenko et al., 2003; Trompouki et al., 2003b). As mentioned previously, CYLD is involved in NF-κB, Wnt/β-catenin and JNK signaling pathway (Reiley et al., 2004; Tauriello et al., 2010; Trompouki et al., 2003b). By using *CYLD* knock-out mice, a recent study shows that in TGF-β-treated T cells, CYLD deficiency causes enhanced TAK1 and p38 mitogen-activated protein kinase activities (Zhao et al., 2011). Accumulation of non-degraded polyubiquitin chains and enhanced activities of SMAD7 in the absence of CYLD led to a study on the putative role of CYLD in the TGF-β signaling (Zhao et al., 2011). This showed that CYLD can bind to SMAD7 and deubiquitinate SMAD7 at Lysine 360 and 374 residues, which are required for the activation of TAK1 and p38 signaling (Zhao et al., 2011).

### USP9X associates with SMAD4

Although SMAD4 is not obligatory for TGF-β signaling, it is required to provide the highest response to signaling. SMAD4 stabilizes SMAD-DNA interaction complexes in the nucleus and also recruits transcriptional coactivators such as histone acetyltransferases to regulatory elements (Wrana, 2009; Yang and Yang, 2010). Compared with other components of the TGF-β/SMAD pathway, SMAD4 possesses a very long half-life and thus is a rather stable protein. Nevertheless, Ectodermin/TRIM33/TIF1γ, a member of TRIM protein family of RING domain E3 ubiquitin ligases, has been suggested to be a determinant of vertebrate gastrulation by targeting SMAD4 for polyubiquitination and degradation (Dupont et al., 2005). This hypothesis was adjusted in a later study by the same group, in which they showed that only the monoubiquitination of SMAD4 is mediated by Ectodermin (Dupont et al., 2009). Lysine 519 of SMAD4 was found to conjugate by Ectodermin with a single ubiquitin molecule in the nucleus, which impairs SMAD4’s binding affinity to R-SMADs. This monoubiquitinated SMAD4 stays in an inhibitory state and regains activity in the cytoplasm once it has been deubiquitinated by FAM/USP9X (Fig. 5) (Dupont et al., 2009). FAM was first discovered in the fly, where FAM stands for *fat facets*. In contrast to what has been shown for FAM/USP9X-mediated deubiquitinating of β-catenin, AF-6, AMPK-related kinase 5 (NUAK1), and microtubule-affinity-regulating kinase 4 (MARK4) (Al-Hakim et al., 2008; Taya et al., 1999; Taya et al., 1998), FAM/USP9X specifically removes the site directed monoubiquitin molecule but not the polyubiquitin chains from SMAD4 (Dupont et al., 2009).

### Other possible DUBs involved in TGF-β signaling

Before the identification and characterization of human DUBs, certain deubiquitinating enzymes were already found to be involved in TGF-β/SMAD signaling, yet not known to act through deubiquitination. Associated molecule with the SH3 domain of STAM (AMSH), a member of JAMMs DUB subfamily, was first identified as a signal-transducing adaptor molecule (STAM) binding protein (Tanaka et al., 1999). AMSH was later found to antagonize the inhibitory effect of SMAD6 on BMP signaling through binding to SMAD6, and did not bind to R-SMAD or Co-SMAD (Itoh et al., 2001). Thus, it will be interesting to examine whether the stimulatory effect of AMSH on BMP signaling is dependent on its DUB activity. Another example is AMSH-2, also a member of the JAMMs subfamily, which has been demonstrated to enhance TGF-β/SMAD signaling when ectopic overexpressed (Ibarrola et al., 2004). Co-immunoprecipitation assays have indicated that AMSH-2 could associate with SMAD2 and SMAD7 (Ibarrola et al., 2004), but also in this case it is not yet known whether the DUB activity of AMSH-2 is required for the enhancement of TGF-β signaling.

## DUBS AS THERAPEUTIC TARGETS

Because of their druggable enzymatic activity, DUBs can be considered as therapeutic targets. Although proteasome inhibitor has been approved for the therapy of multiple myeloma (Hoy, 2013), there are still no DUB inhibitors endorsed for clinical usage. However, multiple studies already revealed such possibilities. As an example, P1130-mediated inhibition of tumor-activated DUBs results in downregulation of antiapoptotic and upregulation of proapoptotic proteins, such as MCL-1 and p53, thereby causing tumor cell apoptosis (Kapuria et al., 2010). A selective inhibitor of the DUB USP14 could be effective against neurodegenerative diseases and myeloma (Lee et al., 2010). Using stereotaxis, direct incubation into brain tumors with PR-619, a broad-spectrum DUB inhibitor, could limit the concentrations of TβR-I and p-SMAD2, in which the effective target is considered to be USP15 (Eichhorn et al., 2012). With the availability of technologies for large scale screening, design and development specific small inhibitor molecules for specific DUBs is required and will be helpful for the generation of novel cancer therapeutics.

## CONCLUSION

The increasing attention for the clinical importance of the TGF-β/SMAD pathway as a tumor promoter makes it more and more worthwhile to search for critical regulators of this pathway as putative therapeutic targets. Since deubiquitinating enzymes can be targeted with drugs, DUBs that control TGF-β/SMAD signaling are emerging as potential targets for cancer therapies (Cohen and Tcherpakov, 2010; Colland, 2010). Several studies utilizing DUB screening methods have provided detailed insights in and mapping of the dynamic functions of ubiquitination in TGF-β/SMAD signaling. Further understanding of the catalytic activity of DUBs, as well as of knowledge on their regulation and substrate specificity, will promote the development of DUB inhibitors as potential anti-cancer drugs. Several DUBs have been identified as driving forces that can trigger and/or enhance tumorigenic TGF-β/SMAD signaling. Among these, promising drug targets are apparently a group of highly-similar DUBs, including USP4, USP11, and USP15. For instance, it would be interesting to develop inhibitors for USP4/11/15 and examine their potentials for anti-invasive and anti-metastatic roles in aggressive human cancers such as breast cancer and glioblastoma.
